# Artemisinin Inhibits Chloroplast Electron Transport Activity: Mode of Action

**DOI:** 10.1371/journal.pone.0038942

**Published:** 2012-06-13

**Authors:** Adyasha Bharati, Monaranjan Kar, Surendra Chandra Sabat

**Affiliations:** 1 Gene Function and Regulation, Stress Biology Laboratory, Institute of Life Sciences, Bhubaneswar, Odisha, India; 2 Department of Botany, Utkal University, Vani Vihar, Bhubaneswar, Odisha, India; Université Joseph Fourier, France

## Abstract

Artemisinin, a secondary metabolite produced in *Artemisia* plant species, besides having antimalarial properties is also phytotoxic. Although, the phytotoxic activity of the compound has been long recognized, no information is available on the mechanism of action of the compound on photosynthetic activity of the plant. In this report, we have evaluated the effect of artemisinin on photoelectron transport activity of chloroplast thylakoid membrane. The inhibitory effect of the compound, under *in vitro* condition, was pronounced in loosely and fully coupled thylakoids; being strong in the former. The extent of inhibition was drastically reduced in the presence of uncouplers like ammonium chloride or gramicidin; a characteristic feature described for energy transfer inhibitors. The compound, on the other hand, when applied to plants (*in vivo*), behaved as a potent inhibitor of photosynthetic electron transport. The major site of its action was identified to be the Q_B_; the secondary quinone moiety of photosystemII complex. Analysis of photoreduction kinetics of *para*-benzoquinone and duroquinone suggest that the inhibition leads to formation of low pool of plastoquinol, which becomes limiting for electron flow through photosystemI. Further it was ascertained that the *in vivo* inhibitory effect appeared as a consequence of the formation of an unidentified artemisinin-metabolite rather than by the interaction of the compound *per se*. The putative metabolite of artemisinin is highly reactive in instituting the inhibition of photosynthetic electron flow eventually reducing the plant growth.

## Introduction

Nature produces a large array of bioactive materials and compounds with unexploited properties. These products have been used more often as structural leads for the discovery and development of natural product based herbicides or pesticides. Often these products have novel sites of action, and even if unsuitable for commercial use, the identification of a new target site is valuable for designing synthetic herbicides [1].

An example of such a natural product is artemisinin, a sesquiterpenoid lactone with a highly reactive endoperoxide bridge from *Artemisia annua L.*, commonly used as a potential antimalarial drug [2]. The compound and several of its structural analogous like, artemisinic acid, arteannuin B, deoxyartemisinin, artesunic acid and sodium artelinate have been reported to possess phytotoxic potential [3]–[8]. It is also shown to inhibit seedling growth in a number of mono and dicotyledonous plants [3]. However, its mode of action and the mechanistic details still remain undeciphered.

Some allelochemicals have been recognized to disrupt the electron transport activity both in chloroplast and mitochondria; including alteration in their membrane architecture [9]. The mitochondrial electron transport chain in yeast has been identified as the major target of this compound as antimalarial agent [10]. Artemisinin, as argued by Li et al. [10] can disrupt the normal electron flow in yeast mitochondria by depolarizing its membrane potential and interacts with NADH-dehydrogenase complex (complex-I).

The chloroplast and mitochondria perform largely a similar kind of function; producing high energy compounds for use in other cellular metabolic activities. Many of the electron transport components between these two organelles are structurally and functionally comparable, like cytochrome system, quinone moieties, ATP-synthesizing complex etc. Further, the chloroplast lamellar system also harbors a mitochondrial homologous NADH-dehydrogenase [11], the chloroplast NADH-dehydrogenase that is engaged in respiratory function of the chloroplast (chloro-respiration).

The discovery of artemisinin interaction with the electron transport chain in yeast and malarial mitochondria systems [10], [12], and considering that the process of photosynthesis, particularly the thylakoid photofunction, being highly susceptible for a large range of herbicides [13], we examined the phytotoxic effect of artemisinin on the chloroplast photoelectron transport activity both under *in vitro* and *in vivo* experimental conditions.

The results from our *in vitro* and *in vivo* studies suggest the presence of two different modes of action of the compound on photosynthetic electron transport. Artemisinin is primarily an energy transfer inhibitor of isolated thylakoid membranes. However, the *in vivo* studies indicate that the compound impairs the thylakoid electron flow as an inhibitor of secondary quinone (Q_B_) of PSII (Photosystem II). The inhibition was determined to be caused by a yet uncharacterized artemisinin- metabolite rather than its direct interaction with thylakoid membrane. Thus, artemisinin may act as a natural prophytotoxin.

## Results

### Inhibitory effect of artemisinin on electron transport activity: an *in vitro* study

**Figure 1:**
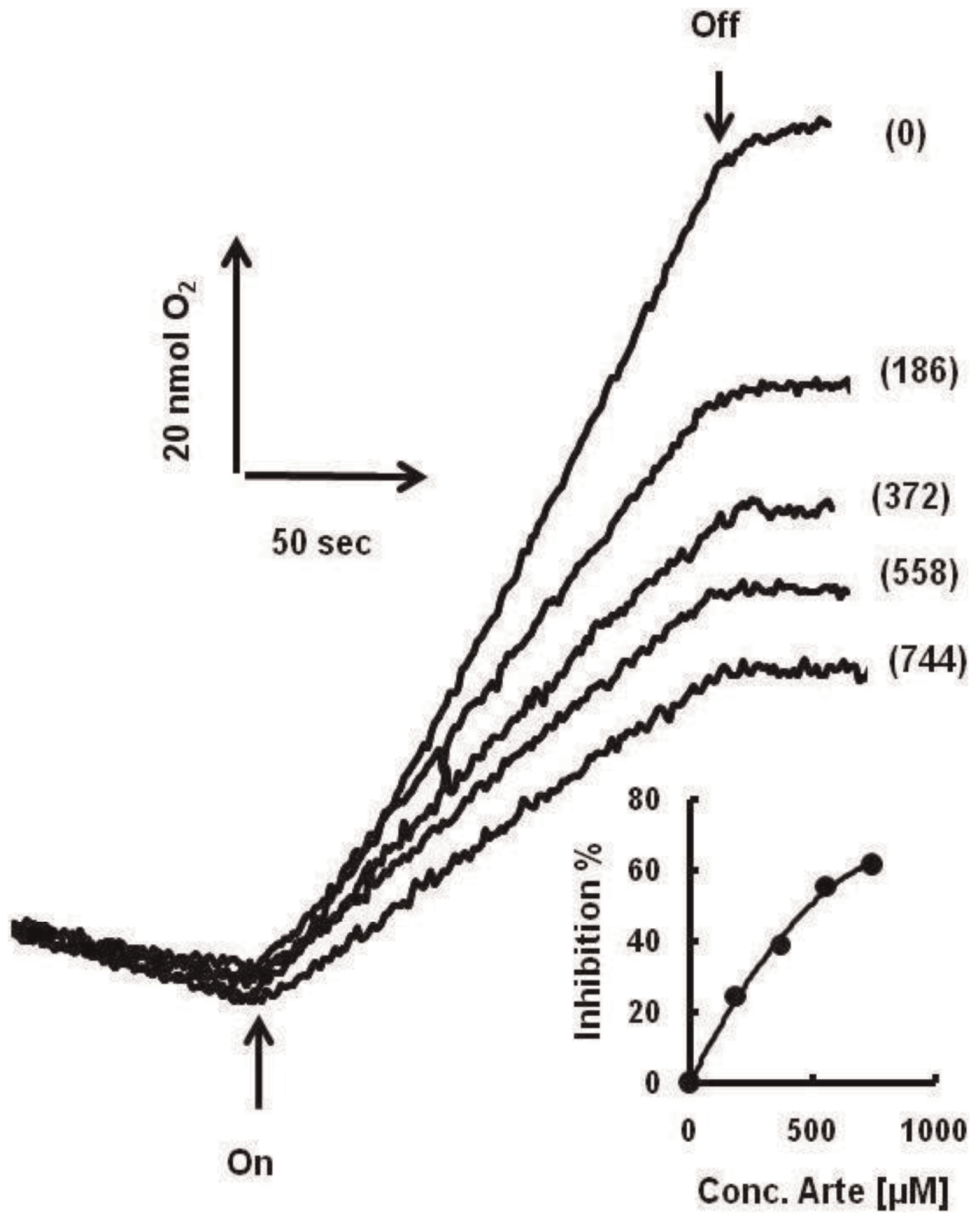
Effect of increasing concentration of artemisinin on FeCN supported O_2_ evolution activity of spinach thylakoids. The concentration of artemisinin used in µM is shown in parenthesis. The inset depicts the increase in percent inhibition of electron transfer rate relative to control with increase in artemisinin concentration. Arrow up, light on; arrow down, light off.

To find out the potency of artemisinin as an inhibitor of thylakoid photofunction, the effect of artemisinin on electron transport rate supported by the transfer of electrons from water to FeCN (potassium ferricyanide) was examined. The extent of inhibition was assessed relative to DMSO (dimethyl sulfoxide) supplemented reaction rates as a control (Fig. 1; trace ‘0’), which was identical to the rate obtained without addition of DMSO. The degree of inhibition was concentration dependent and showed the onset of a saturating tendency around 744 µM; the highest concentration tested in this report. About 65–70% inhibition of control activity was observed at this concentration of artemisinin (inset fig. 1).

**Figure 2:**
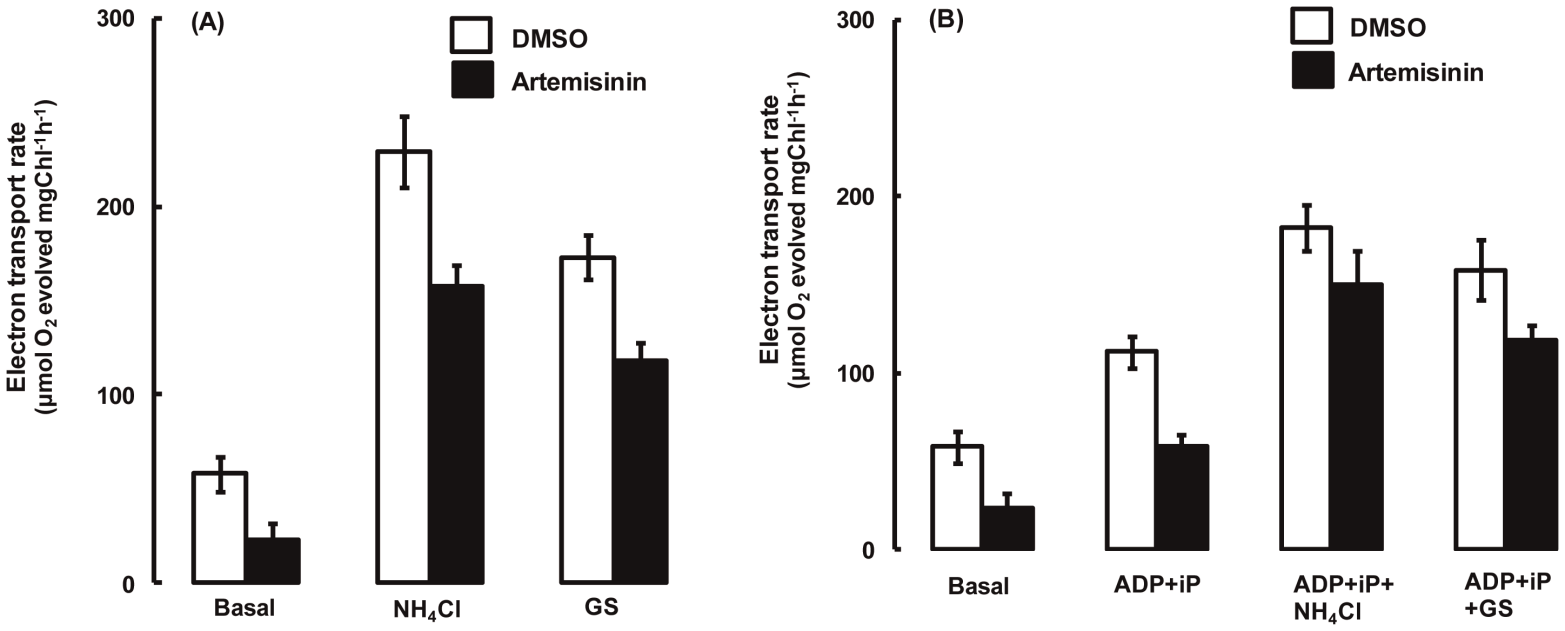
Effect of artemisinin on FeCN supported electron transport in thylakoids under basal, phosphorylating and uncoupled conditions. Artemisinin (744 µM) was added under basal (minus ADP and iP), coupled (plus ADP and iP) and uncoupled (plus NH_4_Cl or GS) conditions with FeCN as terminal electron acceptor. (A) shows electron transport rate as measured in absence of phosphorylating conditions (minus ADP and iP) and (B) shows electron transport rate under phosphorylating conditions (plus ADP and iP). Open bars denote the control (DMSO) and closed bars denote treated (Artemisinin supplemented) rates. Measuring cuvette contained 20 µg equivalent of Chl. thylakoids suspension in 1 ml of the reaction mixture. The error bars show ± SD of electron transport from 5 independent experiments.

The inhibitory effect of the compound was further evaluated under phosphorylating and uncoupled conditions of electron flow. Two different uncouplers known to dissipate the formation of high-energy state through different means were used in this study. The NH_4_Cl (ammonium chloride), an amine type of uncoupler is known to arrest the acidification (chemical potential, ΔpH) of thylakoid lumen [14] and GS (gramicidin-S) is a pore forming ionophore [15] known to collapse the electrical potential (ΔΨ) of high energy state of the membrane. In presence of NH_4_Cl or GS, the extent of artemisinin-mediated (744 µM) inhibition of basal electron transport was reduced by nearly 50% (Fig. 2, A). Addition of ADP (adenosine diphosphate) and iP (inorganic phosphate), as expected, stimulated the basal electron transport rate. However, a decline in the artemisinin-mediated inhibition to 45–50% as against 65–70% in basal conditions was observed under phosphorylating condition (Fig. 2, B). Although the phosphorylating electron transport rate was further stimulated in presence of uncouplers, the toxicity of artemisinin, however drastically reduced it to the tune of 15–20% in presence of NH_4_Cl and about 20–25% with GS (Fig. 2, B).

### Fast chlorophyll (Chl.) *a* (*O-J-I-P*) fluorescence transient and effect of artemisinin: an *in vivo* study

**Figure 3:**
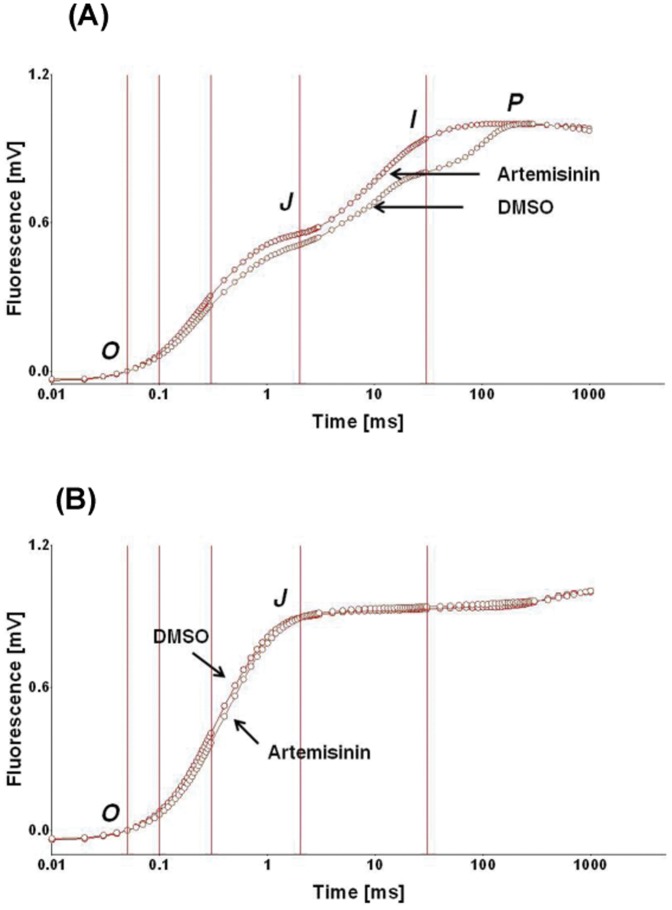
The room temperature Chl. *a O-J-I-P* fluorescence transient of control (DMSO) and artemisinin-treated (Artemisinin) leaves (A), and the effect of DCMU (B). Leaves were floated in DCMU (20 µM) solution for 1 h in complete dark to evaluate its effect in DMSO and artemisinin sprayed leaves. The minor difference in F*_0_* (*O*) and F*_p_* (*P*) obtained was double normalized at F*_O_* and F*_m_* level using biolyzerhp3 software. Each tracing is average plot of nine individual readings. The SD for F*_O_* and F*_m_* in control leaves was 358±9 and 1784±13 and for treated leaves was 368±11 and 1757±19.

To gain insight about the effect of artemisinin *in vivo*, rice plants grown under greenhouse conditions were sprayed with artemisinin as described in materials and methods. Dark adapted leaves when exposed to a strong saturating light (3000 µmol photon m^−2^ s^−1^, red light of 650 nm) for 1 ms, the Chl. *a* fluorescence emission rapidly increases from an initial *O* level to a maximum *P* through transient steps, the *J* and *I*. The *O-J-I-P* uphill rise in fluorescence (F*_O_*, F*_J_*, F*_I_* and F*_P_*) sequence represents the successive reduction of the electron acceptor pools of PSII [16]–[18]. We followed the notations as defined by Strasser et al. [17] to explain the redox significance of these transients. Artemisinin administration induced a small but discernible higher F*_J_* and a higher F*_I_*. The major difference noted due to artemisinin treatment, was the disappearance of the F*_I_ – F_P_* transient (Fig. 3, A). It is known that F*_J_* is very sensitive to changes in the redox state of the PQ-pool. The *F_I_ – F_P_* phase is correlated to the transfer of electrons through PSI as shown by DBMIB (dibromothymoquinone) treatment [19], primarily an antagonist to cytochrome b_563_ but having limited effect on Q_B_ site as well [20], [21]. These alternations in fluorescence characteristics may have arisen due to reduced level of electron flow through PSI, accompanied with changes in the redox state of PQ.

A small but discernible rise in F*_O_ – F_J_* fluorescence intensity suggests the existence of a small effect on Q_A_ reduction in dark adapted leaves. However, a faster rise in Chl. fluorescence from *F_I_* to F*_P_* level in artemisinin treated leaves suggest a slow reduction of Q_B_ resulting in formation of lower pool of PQH_2_ (plastoquinol), which in turn alters the overall redox chemistry of Q_A_ to Q_B_ to PQH_2_ in PSII complex. DCMU (3-(3,4-dichlorophenyl)-1,1-dimethylurea) treatment that inhibits the electron flow from Q_A_ to Q_B_ by binding to Q_B_, results in the transformation of three step fluorescence rise *O-J-I-P* into an *O-J* rise only due to the accumulation of Q_A_^−^. Comparison of the fluorescence induction curves of control and artemisinin treated leaves showed similar sensitivity to DCMU (Fig. 3, B). These results indicate that the electron flow from water to Q_A_ is insensitive to artemisinin toxicity and the compound renders its toxic affect in the span of electron flow beyond Q_A_. The room temperature (25°C) and 77 K fluorescence emission spectral analysis (See Fig. S1, Supporting Information) also suggest that the compound has no effect on the energy transfer from core antenna to PSII reaction centre Chl. [22]. Therefore, to decipher the effect of the compound on the span of electron flow beyond Q_A_, we studied the slow Chl. *a* fluorescence transient, which gives the signature for various photosynthetic events [23].

### Chl. *a* slow fluorescence kinetics

**Figure 4:**
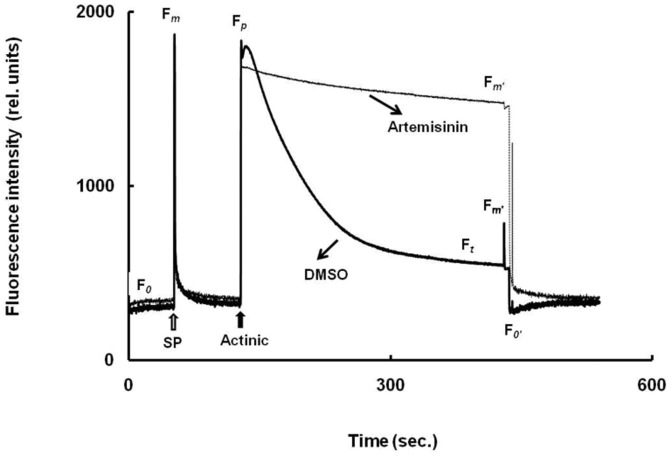
The characteristic room temperature Chl. *a* fluorescence transient of control (DMSO) and artemisinin-treated (Artemisinin) rice leaves. The leaves were washed and dark adapted for 20 min. before subjected for fluorescence measurements. The sequence of light used to evoke the various fluorescence transients are as follows. Minimal fluorescence (F*_O_*) was obtained with low intensity modulated light. Saturating pulse (SP, ≤1 s duration, 18,000 µmol photons m^−2^ s^−1^) was used to give F*_m_* level of fluorescence in darkness and F*_m′_* in light. Actinic light (300 min duration, 615 µmol photons m^−2^ s^−1^) was applied to drive photosynthesis and gives the transient F*_P_*. and F*_t_* level of fluorescence. A short pulse of far–red light was used to get F*_O′_* fluorescence. Each tracing is the average plot of five individual readings. The R_fd_ (fluorescence decline ratio from ‘*p*’ to ‘*t*’ level) value for control and treated sample was noted to be 1.98±0.04 and 0.08±0.006 respectively.

The control rice leaves showed multiphasic Chl. *a* fluorescence induction. Artemisinin treatment to leaves, however, induced an alteration in the relative amplitude of these transients (Fig. 4). A major difference was marked in the quenching of fluorescence from F*_p_* to F*_t_* level. The rise in fluorescence at F*_p_*, in artemisinin treated leaves is followed by a decline to the*‘t’* level but having a much higher amplitude than the control leaf sample. This characteristic phenomenon of alteration in Chl. *a* transient by artemisinin was also observed in spinach and pea (data not shown).

The F*_p_* to F*_t_* decay is manifestly far more complex because of the participation of multiple reactions to quench the fluorescence. The principal causes of this decay relate to gradual re-oxidation of Q_A_ by PSI (photochemical quenching, the qP), the energization of the thylakoid membrane due to proton translocation (non-photochemical or energy dependent quenching, the qE) and possibly the state transition (qT) [24]. The normal deviation of fluorescence decay from F*_p_* to F*_t_* in artemisinin treated leaves as compared to control leave suggest an interruption of electron flow in a site(s) beyond the Q_A_ site of electron acceptance.

The estimated Chl. concentration in both control and artemisinin treated leaves were comparable suggesting that artemisinin treatment had no affect on the Chl. pigment synthesis (See Fig. S2, Supporting Information). We, therefore, further analyzed the electron flow in thylakoid membranes, isolated from artemisinin treated leaves by dissecting the electron transport chain into smaller segments with the use of site specific artificial electron acceptors, donors and inhibitors [25], [26].

### Identification of the target site of artemisinin in the electron transport chain

**Figure 5:**
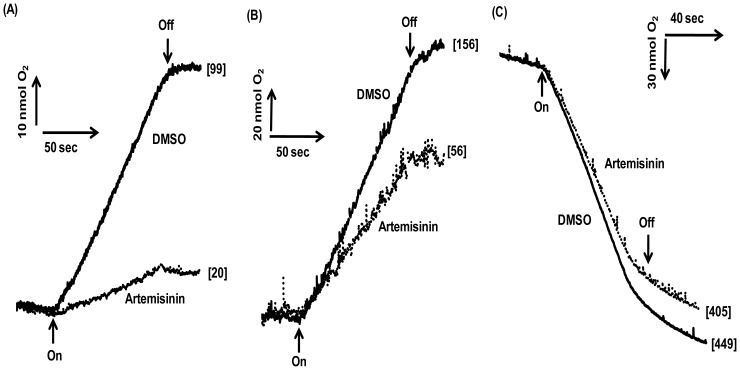
Oxygen exchange activity in whole chain (H_2_O to FeCN, A), PSII (H_2_O to *p*BQ, B) and PSI (DCIPH_2_ to MV, C) catalyzed electron flow in thylakoids isolated from leaves of control (DMSO) and artemisinin-treated (Artemisinin) rice plants. The measuring cuvette contained 20 µg Chl. equivalent thylakoids suspension in 1 ml of the reaction medium. The numbers in parenthesis denote the electron transport rate, expressed as µmol O_2_ evolved (A and B) or consumed (C) mg Chl.^−1^ h^−1^. Arrow up, light on; arrow down, light off.

Thylakoids were isolated from leaves of control and artemisinin sprayed rice plants as described in materials and methods. The FeCN supported whole chain electron transport rate in chloroplasts of artemisinin treated leaves showed a substantially low activity as compared to the rate obtained in control leaf chloroplasts. From various batches of thylakoid preparations, it was inferred that the electron transport rate in chloroplast of treated leaves was reduced by 80–85% as compared to control (Fig. 5, A). Similar extent of inhibition was noted, when FeCN was replaced with MV (methylviologen), another end terminal electron acceptor, which also intercepts electron from the reducing side of PSI (data not shown). Of the total reduction in full chain electron transport, a major reduction in rate of about 65–70% was accounted for *p*BQ (*para*- benzoquinone) supported PSII catalyzed electron transport reaction (Fig. 5, B). The PSI catalyzed electron flow from DCIPH_2_ (reduced-dichlorophenolindophenol) to MV was reduced by 10–15% in artemisinin treated leaves as compared to control rate (Fig. 5, C).

**Figure 6:**
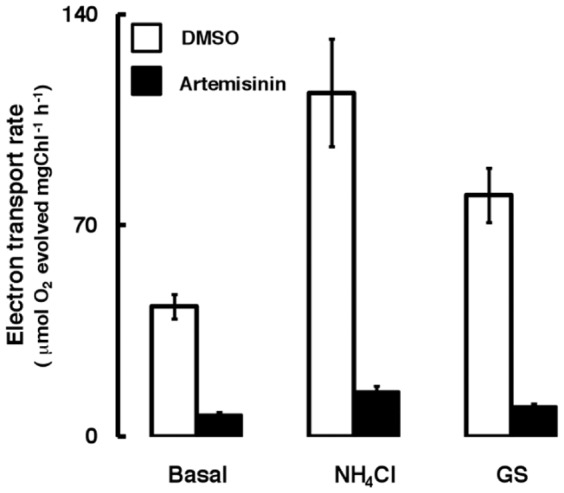
Alteration in the electron transport activity in thylakoids prepared from control (DMSO) and artemisinin-treated (Artemisinin) rice leaves under basal and uncoupled conditions. Thylakoids isolated from leaves of control and treated plants were assayed for alteration in basal and uncoupled (NH_4_Cl or GS) electron transport activities with FeCN as terminal electron acceptor. Measuring cuvette contained 20 µg Chl. equivalent thylakoids suspension in 1 ml of the reaction medium. The number in parenthesis denotes the electron transport rates in µmol O_2_ evolved mg Chl^−1^ h^−1^. Arrow up, light on; arrow down, light off. The error bars indicate ±SD of electron transport (n=3).

Unlike the *in vitro* situation, the inhibitory affect was persistent to a comparable extent both in loosely coupled (basal) and chemically uncoupled thylakoids (Fig. 6), which specifically suggests that, the compound acts primarily as an electron transport inhibitor rather than as an energy transfer inhibitor.

**Figure 7:**
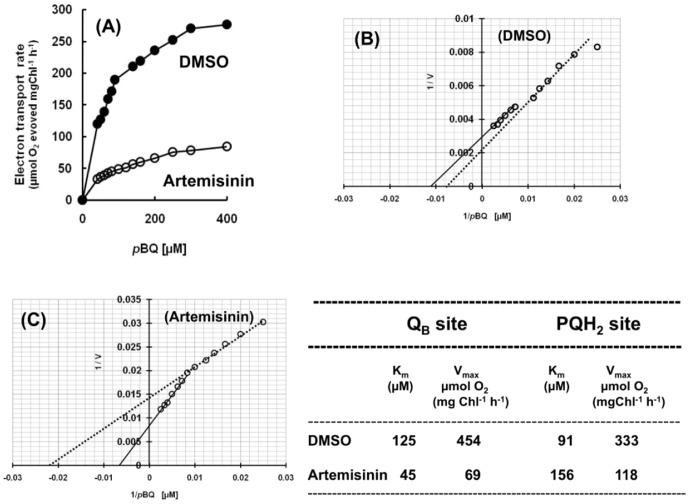
Kinetics of *p*BQ concentration dependent electron transport rate in thylakoids prepared from control (DMSO) and Artemisinin-treated (Artemisinin) leaves. The electron transport rate obtained from the control and treated samples (A) was further analysed for Michaelis-Menten type enzymatic reaction kinetics (B and C). The intersection of the line with the ordinate and the abscissa denotes the inverse values of V_max_ and K_m_ respectively. The values calculated for the respective K_m_ and V_max_ has been shown in the inset table. The *p*BQ titration experiments were carried out in thylakoids isolated both from spinach and rice leaves following similar *in vivo* treatments with artemisinin and the results shown here are from spinach.

**Figure 8:**
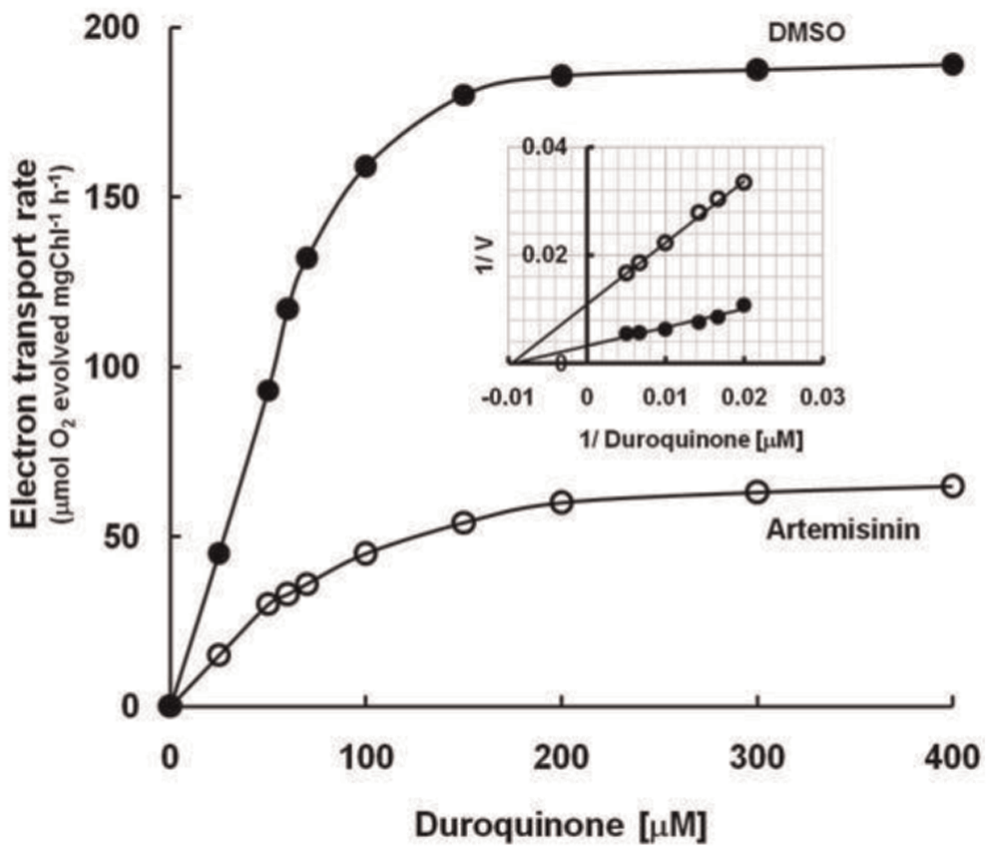
Kinetics of DQ supported electron transport rate in thylakoids prepared from control (DMSO) and Artemisinin-treated (Artemisinin) leaves. The reciprocal plot of the velocity-versus-concentration is shown as inset. The intersection lines with ordinate and abscissa respectively represents the inverse of V_max_ and K_m_.

The lipophilic electron acceptor *p*BQ is known to intercept electrons after Q_A_ in a DCMU sensitive manner, mostly from the plastoquinol pool (PQH_2_) and also from Q_B_ (bound form) site of PSII complex [27], [28]. These sites can be resolved by analyzing the kinetic parameters of electron transport reaction (K_m_ and V_max_), the reciprocal plot of rate (O_2_-evolving activity) against increasing concentrations of *p*BQ (Fig. 7 A, B and C). Similar to an earlier report [27], the plastoquinol pool (PQH_2_) with *p*BQ maintained a high affinity (low K_m_) with low V_max_ against the Q_B_ that possess a low affinity (high K_m_) with high V_max_ value in thylakoids prepared from control leaf (Fig. 7, B). However, artemisinin treatment alters the relative *p*BQ binding affinity (K_m_) and the V_max_ of both these sites in an opposite manner (Fig. 7, C) with a significant reduction in their respective V_max_ amplitude (see the inset table in Fig. 7). The electron transport reaction was further examined as a function of increasing concentration of DQ (duroquinone) that supports PSII catalyzed reaction by intercepting electron only from PQH_2_ pool [27], [29]. Thylakoids prepared from artemisinin treated leaves is found to support a substantially low rate of electron flow from water oxidation complex to DQ as compared to thylakoids of control leaf (Fig. 8). The affinity (K_m_) of DQ for reduced PQ did not change in the thylakoids from the artemisinin treated plants (104–106 µM) despite that, the electron transport rate was 5 times higher in the absence of the sesquiterpenoid lactone (Fig. 8 inset). This indicates that the available pool size of the substrate (PQH_2_) in artemisinin treated leaves is significantly low as compared to control; a condition that might have arisen as or due to inability of conversion of bound quinone to free pool of reduced PQ.

Based on the *in vitro* and *in vivo* experimental conditions, two contrasting situations about the interaction of artemisinin with thylakoid photofunction were observed. These two conditions also differed significantly on the required concentration of the compound to exhibit its maximum inhibitory affect. The effective concentration under *in vivo* condition (see materials and methods) may still be at a lower side considering the penetration efficiency of the compound, spray drift and also the leaf density per plant. However, all our explanations are based on the external sprayed quantity of the compound. Such large discrepancies in artemisinin concentration dependency and differential mode of action under *in vitro* (energy transfer inhibitor) and *in vivo* (electron transport inhibitor) experimental conditions suggest that the inhibition of electron flow under *in vivo* experimental condition may not be a direct consequence of interaction of artemisinin as was the case for *in vitro* situation. Therefore, it is presumed at this stage, that the *in vivo* damage to thylakoid function is possibly routed through some artemisinin-metabolite, rather than direct interaction of the compound *per se*.

### Evidences for the formation of a putative artemisinin-metabolite

In search of an artemisinin-metabolite that could be of importance in instituting the *in vivo* inhibitory affect, we examined the electron transport activity in freshly prepared thylakoids following its incubation with the supernatant prepared from artemisinin treated leaves (see materials and methods).

An inhibition in FeCN supported electron flow was clearly evident in thylakoids incubated with the supernatant obtained from artemisinin treated leaves compared to its counterpart following incubation (See Fig. S3, Supporting Information).

**Figure 9:**
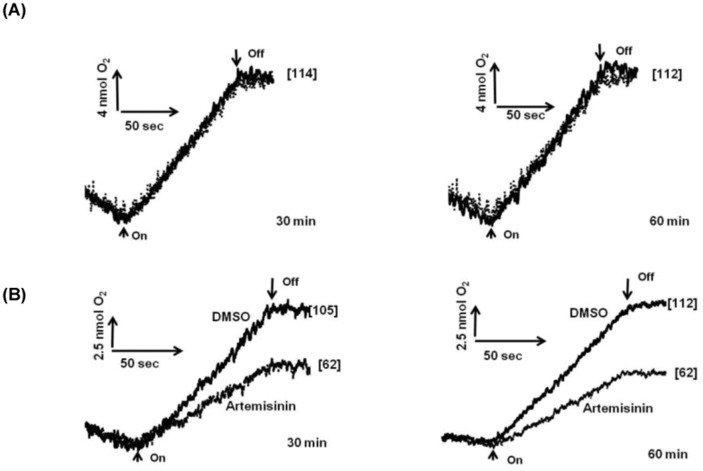
Inhibition of FeCN supported electron transport activity of spinach thylakoid incubated with the supernatant (A) and resuspended pellet (B) obtained following ultracentrifugation. Hundred µg Chl. equivalent control thylakoids suspension were incubated with both the fractions (A, supernatant; B, pelleted fraction) for two different time frames of 30 and 60 min (mentioned below the tracings). FeCN supported electron transport activity was assayed in 1 ml of reaction mixture containing thylakoid suspension equivalent to 20 µg Chl. The number in parenthesis denotes the electron transport rates in µmol O_2_ evolved mg Chl^−1^ h^−1^. Arrow up, light on; arrow down, light off.

The biological activity of the supernatant was checked by fold diluting the supernatant. It was marked that the extent of inhibition was reduced upon dilution of the extract (See Fig. S4, Supporting Information). Further, the supernatant from the 5000×*g* centrifugation was ultracentrifuged as described in materials and methods section. Electron transport rate was analysed by incubating control chloroplasts with both fractions. Samples incubated with the supernatant showed no change in the rates (Fig. 9, A), while about 40–45% decline in electron transport rate was evident in samples incubated with the re-suspended pellet obtained from artemisinin treated leaves (Fig. 9, B).

## Discussion

Much interest is drawn towards the compound, artemisinin, due to its herbicidal activity [3, 4, 30 and 31] in addition to its medicinal use. Although the compound has earlier been tested for growth regulating activity, there is no report on the structural moieties essential for its bioactivity and mechanism of action. We report here the biological activity of artemisinin tested both under *in vitro* and in *vivo* conditions using thylakoid/chloroplast photofunction as assay system. Artemisinin inhibited electron transport rate when added to isolated thylakoids. The assessment of *in vitro* experimental results suggest that artemisinin is effective in developing its maximal inhibitory affect in loosely coupled (basal) followed by fully coupled (phosphorylating) thylakoid electron transport reactions and has a significant low reactivity in chemically uncoupled thylakoids. Therefore, under *in vitro* system the compound behaves more as an energy transfer inhibitor [25] than as a potent inhibitor of electron transport. However, the apparent requirement of a high concentration of artemisinin (744 µM) does not explain the difference in inhibition (of about 65–70%) as shown in this report with that of previous reports on growth regulatory effects in plants as well as in yeast cells. At 33 µM artemisinin, inhibited the germination of lettuce and annual wormwood, and growth of roots and shoots in lettuce, redroot pigweed, pitted morning glory, annual wormwood and common purslane [6]. Also at a concentration of 80 µM, artemisinin arrests the growth of yeast cells [10]. An attempt was, therefore, made here to reassess the effect of artemisinin on the photoelectron transport activity in thylakoids isolated from leaves of plants externally treated with the compound.

In order to elucidate the relationship between the concentration and biological activity of the compound, it was necessary to use a test system that required minimal amount of the compound and still showed biological activity. Therefore, we sprayed rice plants with artemisinin at concentrations 1.86 µmol of artemisinin/plant (see materials and methods) and reassessed its toxicity. Chl. *a* fluorescence transient was measured to evaluate the effect of artemisinin on the photochemical efficiency of PSII. The *O-J-I-P* transient depicts the rate of reduction kinetics of various components of PSII. Addition of artemisinin resulted in the disappearance of F*_I_*-F*_P_* transient which has been interpreted as the result of slower reduction of Q_B_ due to presence of a smaller plastoquinol pool in comparison to the control. A small but relative increase in variable fluorescence as a function of artemisinin was indicative of a loss in Q_A_^−^ re-oxidation capacity. Further, the presence of comparable DCMU mediated induction of fluorescence emission suggest that artemisinin has no affect on the span of electron flow from water to Q_A_. The above conclusion was further supported from the slow fluorescence transient obtained following *in vivo* exposure of rice leaves to artemisinin. The ground level fluorescence (F*_O_*) and the maximal fluorescence (F*_m_*) yield did not change appreciably, but there were pronounced changes in the relative decay from F*_P_* to F*_t_*. This deviation represents the slow rate of electron flow from Q_B_ to intersystem electron transport components.

To investigate the contribution of artemisinin towards the inhibition of electron flow, thylakoids were isolated from leaves of control and artemisinin sprayed plants and evaluated for their respective electron transport rate in the presence of site specific electron donors and acceptors, and also uncouplers. This sesquiterpene lactone was found to behave as a strong inhibitor of electron flow, potentially a specific inhibitor to Q_B_ functioning. The Q_B_ quinione moiety in thylakoid membrane remains housed in a 32 kDa peptide (D1 polypeptide) of PSII core complex, which otherwise is also known as the herbicidal binding protein. Many synthetic products like DCMU, atrazine and also natural compounds such as fisherellin, grandinol, sorgoleone etc. [32], [1] have been shown to interact with this protein impairing the electron flow beyond Q_A_. The detailed mechanism of interaction at molecular level, either by forming an inhibitory complex with the protein (chemical modification) or with the quinone moiety (steric hindrance) for some of these compounds has also been deciphered [32].

Both the *in vitro* and *in vivo* scenarios presented different outcomes in our study, thus emphasizing that the mode of action of the compound in plant system (*in vivo*) is not a direct interaction of the compound with the thylakoid electron transport event, but illustrates the presence of a putative artemisinin-inhibitory-metabolite. The metabolite on homogenization comes out of the cellular component and is present in the supernatant. The supernatant was tested for inhibition in normal chloroplasts. Results from incubation studies show that the supernatant obtained from homogenization of artemisinin treated leaves is capable of inhibiting electron flow to about 65–70% (See Fig. S3, Supporting Information). Further, the metabolite is confirmed to be particulate in nature as ultracentrifugation data reveal its presence in the pellet. The pelleted fraction is mostly comprised of cell organelles like mitochondria, golgi and other membrane structures, but less of nuclei and plastids. Here, further experiments are needed to exactly decipher the organelle specific location and association of the artemisinin-metabolite. However, the compound does get degraded in the cell free extract, as storage of the supernatant leads to decline in its inhibitory effect (chloroplasts incubated with supernatant stored for 24 h at 4°C does not show any inhibition). We also observed that long term administration of artemisinin leads to the death of plant (See Fig. S5, Supporting Information).

For artemisinin, as described in this report, we have so far been successful in only identifying the mode (site) of its action on the photosynthetic electron transport. The significance of addressing the action of a natural phytotoxin often lies in the novelty of their site and mechanism of action. Although, as mentioned earlier their use may not be commercially viable, the identification of a new target site is always valuable in the design of new synthetic herbicides. Therefore, artemisinin could be selected as a suitable lead for development as an effective herbicide and/or for the design of new biodegradable agent with agrochemical applications [1]. However in absence of the structural features of the proposed putative artemisinin-inhibitory-metabolite, the intrinsic mechanism of its interaction either with the protein or with the Q_B_ moiety of PSII reaction centre remain to be worked out to fully understand the mechanism of action of artemisinin.

## Materials and Methods

### Materials

Purified artemisinin was obtained as gift from Ipca Laboratories Ltd., (India). DMSO was used to make stock solution of artemisinin. The electron acceptors like FeCN, DCIP, MV, DQ, *p*BQ were obtained from Sigma. The electron transport inhibitor DCMU from Sigma was recrystallized from 95% ethanolic solution. The buffers used were also procured from Sigma. Other chemicals used in the experiment were of analytical grade.

### Plant materials

Fresh spinach (*Beta palonga*) was obtained from local market. Rice (*Oryza sativ*a var. Safari) plant was grown in soil under greenhouse conditions of light, temperature and humidity. The leaves were thoroughly washed in distilled water before using them for experimental purpose.

### Plant growth and artemisinin treatment

Twenty one day old rice plants were sprayed with artemisinin twice at a 24 h interval. One mililiter of 74.4 mM artemisinin in 1% (V/V) DMSO, 0.1% (V/V) TWEEN-20 was diluted 100 fold with water and used to treat 40 plants (∼1.86 µmol of artemisinin/plant). Control plants were sprayed with the same solution without artemisinin. The 4^th^ (quaternary) leaf was harvested 20 h after the 2^nd^ spray and used for all experiments. The individual effect of the surfactant and DMSO-plus-surfactant was first evaluated against water control. Since no effect was obtained with these variations, we have routinely used the DMSO plus the surfactant sprayed leaves as control against artemisinin treated leaves and in figures the same has been depicted as DMSO (control).

### Chlorophyll *a* fluorescence measurement in intact leaves

The room temperature Chl. *a* fluorescence technique was used to study the photosynthetic activity in intact leaf samples. The fast Chl. *a* transients (*O-J-I-P*) in millisecond (ms) time span was studied with plant efficiency analyzer (Handy PEA fluorimeter) following Strasser et al. [17], while the relative thylakoid electron transport, membrane energization and carbon fixation activities were evaluated from slow fluorescence transients, obtained using FMS2 instrument (Hansatech) as per the detail outlined in Baker [23]. The readouts from Handy PEA were analyzed using biolyzerhp3 software.

### Isolation of thylakoid membrane

Thylakoids were isolated from spinach and rice leaves following [33] with some modifications. Leaves were homogenized in cold grinding buffer [50 mM tricine-NaOH (pH 7.5), 400 mM sucrose, 5 mM MgCl_2_ and 5 mM KCl]. The homogenate was filtered through Miracloth and the filtrate was centrifuged at 5000×*g* for 5 min. The chloroplast pellet was washed in 5 mM tricine-NaOH (pH 7.5) buffer containing, 400 mM sucrose and 2 mM MgCl_2_ and centrifuged at 300×*g* for 1 min. The supernatant was re-centrifuged at 5000×*g* for 5 min. The pellet (thylakoids) was finally taken up in a minimal volume of suspending buffer [20 mM tricine-NaOH (pH 7.5), 100 mM sucrose and 5 mM MgCl_2_]. Chlorophyll concentration in methanolic extract was measured spectrophotometrically [34].

### Electron transport measurement

Photosystem II, PSI and whole chain (PSII plus PSI) catalyzed electron transport activities were measured as oxygen (O_2_) evolution or uptake in a Clarke type O_2_ electrode assembly (Hansatech) at 22±1°C using site-specific artificial electron acceptors, donors, and inhibitors [25], [26]. The light was rate-saturating (500 µE m^−2^ s^−1^). Phosphorylating electron transport was measured with addition of 1 mM ADP and 5 mM K_2_HPO_4_ (iP) to basal electron transport components (25 mM hepes-NaOH (pH-7.5), 100 mM sucrose, 5 mM MgCl_2_ and 20 µg Chl. ml^−1^). NH_4_Cl and GS were used at a final concentration of 5 mM and 10 µM respectively as and when required. The whole chain electron flow was assayed in presence of 2 mM FeCN as terminal electron acceptor of PSI. *p*BQ and DQ (400 µM) were used to measure PSII catalyzed electron flow. To measure PSI catalyzed electron flow, DCIPH_2_ (1 mM DCIP plus 2 mM ascorbate) was used as electron donor to cytochrome *b_6_/f* region and 1 mM MV as transient electron acceptor. The PSI reaction mixture additionally contained 2 mM sodium azide (catalase inhibitor) and 10 µM DCMU (PSII inhibitor).

All experiments were repeated with 3 to 4 individual batches of thylakoid preparations. Experiments in intact plant samples were also made from different batches of plants. Although the absolute values of electron transport activity among various batches varied between 10–12%, the phenomenological observations were consistent.

### Putative artemisinin-metabolite analysis

Leaf tissue (700 mg) each from control and artemisinin treated rice leaves were homogenized in 6 ml of the grinding buffer, filtered and the filtrate was centrifuged at 5000×*g* for 5 min. Supernatant was kept in ice for further experimentation. Freshly isolated thylakoids (100 µg Chl. equivalent) from spinach were incubated with 1 ml of the supernatant obtained from artemisinin treated leaves for different time intervals (supernatant obtained from homogenized tissue sprayed with DMSO were taken as control for all experiments). The thylakoids were then spun down (5000×*g* for 5 min), washed once with washing buffer, resuspended in suspension buffer and assayed for their respective electron transport activities. In order to further characterize the nature of the inhibitory-metabolite, the supernatant from 5000×*g* centrifugation was ultracentrifuged at 1,81,700×*g* (50,000 rpm in the Sorvall TFT 80.4 rotor) for 1 h at 4°C. The supernatant was collected carefully and the pellets were resuspended in 1 ml of the suspending buffer. Isolated chloroplasts (100 µg Chl. equivalent) from spinach were incubated in the fractions for 30 and 60 min. The pelleted thylakoids, following the treatment, were assayed for whole chain electron transport activity with FeCN as electron acceptor.

## Supporting Information

Figure S1 Room temperature (25°C) and 77 K fluorescence spectra of isolated thylakoid from control (DMSO) and artemisinin treated (Artemisinin) leaves. The Chl. concentration was 3 µg ml^−1^. 40% glycerol was used in the chloroplast suspension medium for low temperature (77 K) spectral measurements.
(DOC)

Figure S2 Chlorophyll quantification in plants sprayed with DMSO and artemisinin. Leaves (20 mg fresh weight) were cut into small pieces and homogenized in cold methanol. The methanolic extract was used for estimation of Chl. Open bars denote Chl. content of control (DMSO) and closed bars denote Chl. content of treated (Artemisinin) plants. The graph shows an average of 5 individual readings with ± SD as error bars. Inset depicts the Chl. (*a/b*) ratio in both control and artemisinin sprayed leaves.
(DOC)

Figure S3 Analysis of inhibition of electron transport activity by putative artemisinin-metabolite. A, B, C and D respectively depicts the incubation times of 10, 20, 30, 60 min. The extent of inhibition was marked to be a time dependent phenomenon and a maximum inhibition of 65–70% was observed following 30 min of incubation. Increasing the incubation period to 60 min did not alter the extent of this inhibition. The numbers in parenthesis denote the electron transport rate (µmol O_2_ evolved mg Chl.^−1^ h^−1^) measured in presence of FeCN as electron acceptor. Using lesser amount of buffer during homogenization led to a much concentrated solution that failed to show any measurable O_2_ exchange activity in thylakoids incubated with supernatant from leaves of artemisinin treated plants. Hence, we routinely used the mentioned ratio (see materials and methods) of tissue to grinding buffer for preparing the tissue supernatant. Arrow up, light on; arrow down, light off.
(DOC)

Figure S4 Analysis of biological activity of supernatant. The biological activity of supernatant was checked by fold dilution. With increase in dilution, the extent of inhibition was reduced, as measured against the undiluted supernatant.
(DOC)

Figure S5 Long term effect of artemisinin administration to rice plant. The plants were treated with artemisinin for 5 consecutive alternate day sprays. It resulted in complete death of the plants while the control plants (DMSO sprayed) continued to grow without symptoms of senescence.
(DOC)
