# Supplementary material for: Artemisinin Inhibits Chloroplast Electron Transport Activity: Mode of Action
Source: PLoS One. 2012 Jun 13;7(6):e38942. doi: 10.1371/journal.pone.0038942 (PMC3374801; doi:10.1371/journal.pone.0038942)
Supplement: Figure S1 — Room temperature (25°C) and 77 K fluorescence spectra of isolated thylakoid from control (DMSO) and artemisinin treated (Artemisinin) leaves. The Chl. concentration was 3 µg ml−1. 40% glycerol was used in the chloroplast suspension medium for low temperature (77 K) spectral measurements. (DOC) [file pone.0038942.s001.doc]

**Figure S1.** Room temperature (25°C) and 77 K fluorescence spectra of isolated thylakoid from control (DMSO) and artemisinin treated (Artemisinin) leaves. The Chl. concentration was 3µg ml^-1^. 40% glycerol was used in the chloroplast suspension medium for low temperature (77 K) spectral measurements.
