# Supplementary material for: Artemisinin Inhibits Chloroplast Electron Transport Activity: Mode of Action
Source: PLoS One. 2012 Jun 13;7(6):e38942. doi: 10.1371/journal.pone.0038942 (PMC3374801; doi:10.1371/journal.pone.0038942)
Supplement: Figure S2 — Chlorophyll quantification in plants sprayed with DMSO and artemisinin. Leaves (20 mg fresh weight) were cut into small pieces and homogenized in cold methanol. The methanolic extract was used for estimation of Chl. Open bars denote Chl. content of control (DMSO) and closed bars denote Chl. content of treated (Artemisinin) plants. The graph shows an average of 5 individual readings with ± SD as error bars. Inset depicts the Chl. (a/b) ratio in both control and artemisinin sprayed leaves. (DOC) [file pone.0038942.s002.doc]

**Figure S2.** Chlorophyll quantification in plants sprayed with DMSO and artemisinin**.** Leaves (20 mg fresh weight) were cut into small pieces and homogenized in cold methanol. The methanolic extract was used for estimation of Chl. Open bars denote Chl. content of control (DMSO) and closed bars denote Chl. content of treated (Artemisinin) plants. The graph shows an average of 5 individual readings with ± SD as error bars. Inset depicts the Chl. (*a/b*) ratio in both control and artemisinin sprayed leaves.
