# Supplementary material for: Artemisinin Inhibits Chloroplast Electron Transport Activity: Mode of Action
Source: PLoS One. 2012 Jun 13;7(6):e38942. doi: 10.1371/journal.pone.0038942 (PMC3374801; doi:10.1371/journal.pone.0038942)
Supplement: Figure S3 — Analysis of inhibition of electron transport activity by putative artemisinin-metabolite. A, B, C and D respectively depicts the incubation times of 10, 20, 30, 60 min. The extent of inhibition was marked to be a time dependent phenomenon and a maximum inhibition of 65–70% was observed following 30 min of incubation. Increasing the incubation period to 60 min did not alter the extent of this inhibition. The numbers in parenthesis denote the electron transport rate (µmol O2 evolved mg Chl.−1 h−1) measured in presence of FeCN as electron acceptor. Using lesser amount of buffer during homogenization led to a much concentrated solution that failed to show any measurable O2 exchange activity in thylakoids incubated with supernatant from leaves of artemisinin treated plants. Hence, we routinely used the mentioned ratio (see materials and methods) of tissue to grinding buffer for preparing the tissue supernatant. Arrow up, light on; arrow down, light off. (DOC) [file pone.0038942.s003.doc]

**Figure S3.** Analysis of inhibition of electron transport activity by putative artemisinin-metabolite. A, B, C and D respectively depicts the incubation times of 10, 20, 30, 60 min. The extent of inhibition was marked to be a time dependent phenomenon and a maximum inhibition of 65-70 % was observed following 30 min of incubation. Increasing the incubation period to 60 min did not alter the extent of this inhibition. The numbers in parenthesis denote the electron transport rate (µmol O_2_ evolved mg Chl.^-1^ h^-1^) measured in presence of FeCN as electron acceptor. Using lesser amount of buffer during homogenization led to a much concentrated solution that failed to show any measurable O_2_ exchange activity in thylakoids incubated with supernatant from leaves of artemisinin treated plants. Hence, we routinely used the mentioned ratio (see materials and methods) of tissue to grinding buffer for preparing the tissue supernatant. Arrow up, light on; arrow down, light off.
