# Supplementary material for: Artemisinin Inhibits Chloroplast Electron Transport Activity: Mode of Action
Source: PLoS One. 2012 Jun 13;7(6):e38942. doi: 10.1371/journal.pone.0038942 (PMC3374801; doi:10.1371/journal.pone.0038942)
Supplement: Figure S4 — Analysis of biological activity of supernatant. The biological activity of supernatant was checked by fold dilution. With increase in dilution, the extent of inhibition was reduced, as measured against the undiluted supernatant. (DOC) [file pone.0038942.s004.doc]

**Figure S4.** Analysis of biological activity of supernatant**.** The biological activity of supernatant was checked by fold dilution. With increase in dilution, the extent of inhibition was reduced, as measured against the undiluted supernatant.
