# Supplementary material for: Artemisinin Inhibits Chloroplast Electron Transport Activity: Mode of Action
Source: PLoS One. 2012 Jun 13;7(6):e38942. doi: 10.1371/journal.pone.0038942 (PMC3374801; doi:10.1371/journal.pone.0038942)
Supplement: Figure S5 — Long term effect of artemisinin administration to rice plant. The plants were treated with artemisinin for 5 consecutive alternate day sprays. It resulted in complete death of the plants while the control plants (DMSO sprayed) continued to grow without symptoms of senescence. (DOC) [file pone.0038942.s005.doc]

**Figure S5.** Long term effect of artemisinin administration to rice plant. The plants were treated with artemisinin for 5 consecutive alternate day sprays. It resulted in complete death of the plants while the control plants (DMSO sprayed) continued to grow without symptoms of senescence.
